# Supplementary figures and images for: Nutrient acquisition drives Edwardsiella tarda pathogenesis in necrotizing soft tissue infection
Source: mSystems. 2026 Jan 16;11(2):e01657-25. doi: 10.1128/msystems.01657-25 (PMC12911395; doi:10.1128/msystems.01657-25)

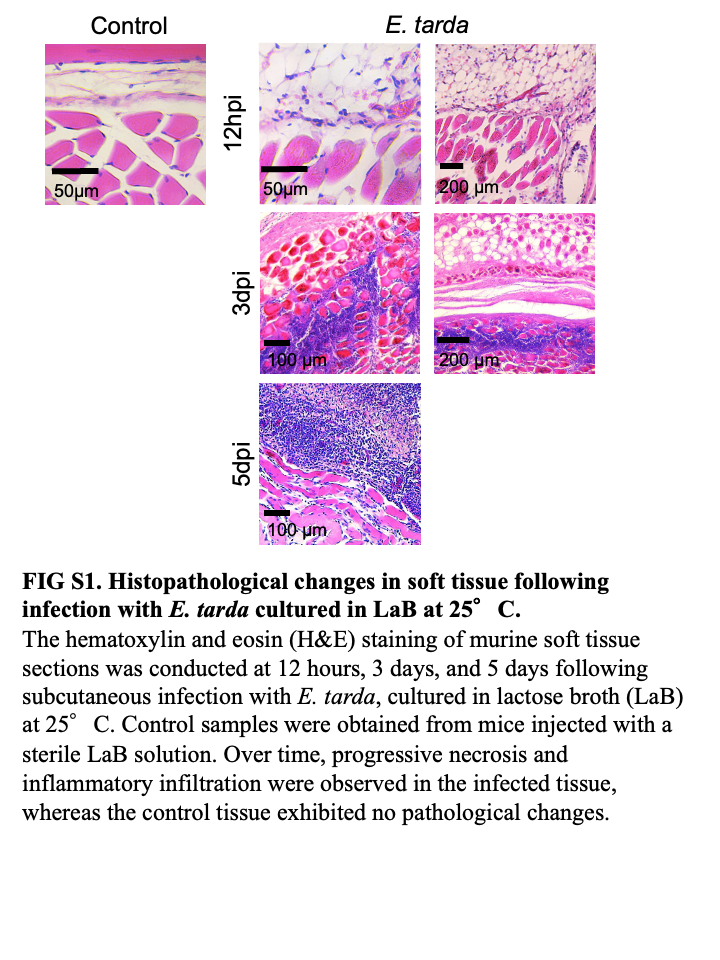

Supplement: Fig. S1 — Histopathological changes in soft tissue following infection with E. tarda cultured in LaB at 25°C. [file msystems.01657-25-s0001.tiff]

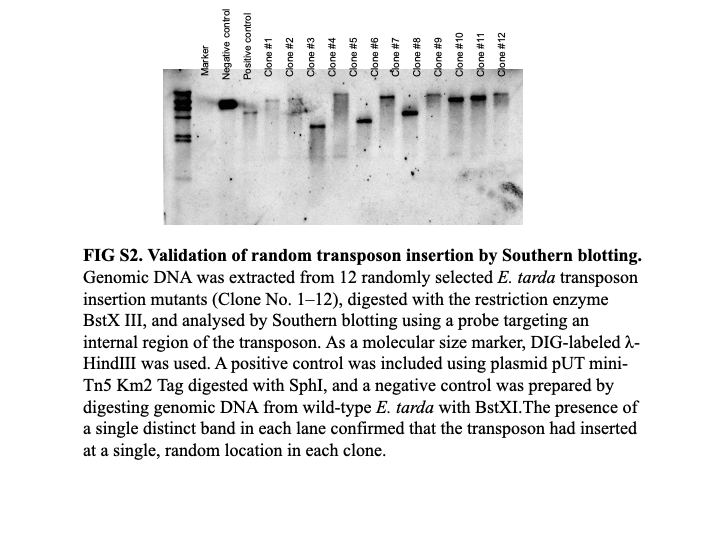

Supplement: Fig. S2 — Validation of random transposon insertion by Southern blotting. [file msystems.01657-25-s0002.tiff]
